# External validation of binary machine learning models for pain intensity perception classification from EEG in healthy individuals

**DOI:** 10.1038/s41598-022-27298-1

**Published:** 2023-01-05

**Authors:** Tyler Mari, Oda Asgard, Jessica Henderson, Danielle Hewitt, Christopher Brown, Andrej Stancak, Nicholas Fallon

**Affiliations:** Department of Psychology, Institute of Population Health, University of Liverpool, 2.21 Eleanor Rathbone Building, Bedford Street South, Liverpool, L69 7ZA UK

**Keywords:** Cognitive neuroscience, Computational neuroscience

## Abstract

Discrimination of pain intensity using machine learning (ML) and electroencephalography (EEG) has significant potential for clinical applications, especially in scenarios where self-report is unsuitable. However, existing research is limited due to a lack of external validation (assessing performance using novel data). We aimed for the first external validation study for pain intensity classification with EEG. Pneumatic pressure stimuli were delivered to the fingernail bed at high and low pain intensities during two independent EEG experiments with healthy participants. Study one (n = 25) was utilised for training and cross-validation. Study two (n = 15) was used for external validation one (identical stimulation parameters to study one) and external validation two (new stimulation parameters). Time–frequency features of peri-stimulus EEG were computed on a single-trial basis for all electrodes. ML training and analysis were performed on a subset of features, identified through feature selection, which were distributed across scalp electrodes and included frontal, central, and parietal regions. Results demonstrated that ML models outperformed chance. The Random Forest (RF) achieved the greatest accuracies of 73.18, 68.32 and 60.42% for cross-validation, external validation one and two, respectively. Importantly, this research is the first to externally validate ML and EEG for the classification of intensity during experimental pain, demonstrating promising performance which generalises to novel samples and paradigms. These findings offer the most rigorous estimates of ML’s clinical potential for pain classification.

## Introduction

Establishing an accurate assessment of subjective pain intensity is imperative for the diagnosis, prognosis and treatment of chronic pain conditions^1,2^. Current pain assessment methods are contingent on self-report measures, which are not appropriate for individuals who are unable to communicate their pain precisely or entirely, such as those with dementia^3,4^, disorders of consciousness (e.g., coma)^3,5^, cognitive impairments^3,6^, non-verbal individuals (e.g., non-communicative palliative care patients)^3,7^, and children (e.g., infants and neo-natal populations)^3,8^. Furthermore, pain is an inherently subjective and multifaceted sensory process, which is challenging to measure objectively^1,4^. Taken together, the complexity of accurate pain assessment, particularly in populations with a reduced capacity for self-report, demonstrates the necessity for improved objective evaluation methods.

Recent endeavours to mitigate the necessity of self-report methods have attempted to elucidate biological markers of pain intensity using neuroimaging (see^9,10^). ML analysis of neuroimaging data further enables the identification of pain intensity biomarkers. ML refers to algorithms that identify and learn patterns from data to make predictions on novel inputs without being explicitly programmed, which is achieved using optimisation, statistical and probabilistic techniques^11–13^. The primary aim of supervised ML is to identify a function, *f*, that achieves the best mapping of an input *X*, to an output *Y* (see Eq. 1)^13,14^. To identify the optimal function, supervised ML algorithms are trained using labelled data to minimise a loss (error) function by altering internal parameters^15,16^. Following training, the model is evaluated on novel data to assess its generalisability.

1 $$f :X \to Y$$

Pain-related neural activation forms a distributed network (e.g., neurologic signature^17^)^18^, and includes primary (SI) and secondary somatosensory cortex (SII), insula, thalamus, anterior and midcingulate cortex, prefrontal cortex, amygdala, middle frontal gyrus, cerebellum and brainstem^19–21^. In addition, different regions encode specific characteristics of pain; SI and SII encode temporal, spatial and intensity features^22,23^, whilst the insula contributes to encoding stimulus salience^24^.

Regarding EEG, pain modulates cortical oscillations in theta, alpha, beta and gamma frequency bands across various cortical sites including frontal, central, parietal, temporal and occipital regions^25–27^. Altered theta oscillations (4–7 Hz) are commonly observed in resting state EEG of individuals with chronic pain^25^, e.g., in fibromyalgia syndrome patients^28^. Moreover, augmented theta oscillations have been observed during pain and touch stimulation over central and parietal regions, with larger increases during painful stimulation^29^. Additionally, tonic pain stimulation is associated with decreased alpha and increased beta band power (see^25–27^ for reviews). Research has demonstrated decreased global alpha and increased beta band power in response to tonic cold pain stimulation^30^. Source analysis identified pain-related oscillations predominantly in prefrontal cortex, SI, SII, insular cortex and cingulate cortex^30^. Recently, peak alpha frequency has been shown to reliably predict pain sensitivity^31,32^. Finally, gamma oscillations over SI have been shown to predict subjective pain intensity^33,34^ and stimulus intensity^33^. Consequently, EEG features may be used as a neural marker of pain intensity.

Previous research has successfully implemented ML to identify pain intensity using EEG^10^. Our recent systematic review demonstrated that EEG and ML could discriminate the presence or absence of pain with accuracies between 82.73 and 95.33% and predict pain intensity with accuracies between 62 and 100%^10^. Moreover, ML classified low and high pain intensity, with the best-performing models achieving cross-validated accuracies of up to 62%, 69.20%, 70.36%, 83.50%, 86.30% and 89.58%^35–40^. Overall, these findings demonstrate the potential of ML for identifying pain intensity in healthy individuals, with all studies performing significantly better than chance.

Specifically, Misra and colleagues^40^ used a Gaussian support vector machine (SVM) to successfully classify low and high pain using theta and gamma power over the medial prefrontal region and lower beta power over the contralateral sensorimotor region. Moreover, a naïve Bayes classifier has been used to discriminate pain intensity using single-trial laser-evoked potentials^39^. That study found that low and high pain could be classified with accuracies greater than 80% for both within-subject and cross-subject classifications. In the same study, the continuous pain rating (0–10) was predicted with a mean absolute error of less than 2 for both within-subject and cross-subject levels. Furthermore, similar research used EEG and a random forest (RF) to classify pain intensity into 10 classes (1–10); achieving accuracies close to 90% for both within-subject and cross-subject classifications^41^. Interestingly, the study evaluated the relative contributions of each frequency band to the classification performance and found that all frequency bands were important to the classification (delta, theta, alpha, beta, gamma), with gamma being the most important to the classification performance. Therefore, including a diverse array of frequency bands and electrode locations would likely achieve optimal classification performance.

Despite previous research demonstrating promising performance, it is unclear if these models will successfully generalise to new samples. No studies in the existing literature have reported external validation; the process of evaluating a model using novel data, collected at a different time, geographical location, or using a different experimental paradigm^42^. Previous research only assessed cross-validation performance. Cross-validation involves partitioning a single dataset into training and testing sets, such that the test set is used to estimate the model’s prediction error^43^. Although cross-validation is essential in model development, it can lead to overly-optimistic estimates of model performance and overfitting (where the model learns idiosyncrasies in the training set, which diminishes performance on novel data)^44–47^. Consequently, the previous research findings are potentially inflated and may not be generalisable^10^, which is insufficient evidence for clinical translation^48,49^. However, a recent study found that pain-free sensorimotor peak alpha frequency could correctly classify pain-sensitive individuals using an external validation paradigm^32^, providing evidence that EEG and ML could be effectively combined to identify pain outcomes. Nevertheless, external validation has never been attempted for investigations of pain intensity classification.

The present study aimed to be the first to externally validate ML for EEG pain intensity classification, through a robust two-step process. Given the paucity of external validation research, we aimed (1) to train ML classifiers on EEG data to predict pain intensity (low, high) and evaluate the cross-validation performance, (2a) to externally validate the classifiers on data collected from a novel sample at a different time, which used identical stimulation and (2b) to externally validate the models on data obtained at a different time, which used different stimulation parameters. We conducted this multistep validation to thoroughly assess model performance and generalisability using seven well-researched supervised ML models. We hypothesised that all ML algorithms would classify pain intensity with performance metrics (accuracy and area under the receiver operating characteristics curve, hereinafter AUC) greater than chance level (≈ 50%) on (1) cross-validation and (2a) external validation one (same stimulation parameters) and (2b) external validation two (different stimulation parameters).

## Methods

Two independent experiments, separated by approximately 4 months, were conducted. Study one was used for training and cross-validation, whilst study two was used for external validation. Moreover, study two included external validation one, which used the same stimulus parameters as study one, and external validation two, which used different parameters (external validation datasets were collected simultaneously). Both studies were processed using a similar pipeline but were managed independently to prevent data leakage^50^, which could have biased the external validation. The classification was performed across all trials, pooled from every participant. The EEG data is freely available through the Open Science Framework (https://osf.io/uqt9z/).

### Participants

Forty healthy subjects (29 female) aged between 18 and 37 years were recruited across both studies using opportunity sampling. Twenty-five participants (19 female) aged 18–37 years (Mean = 23.64 years, SD = 4.04) completed study one, whilst 15 participants (10 female) aged between 19 and 28 years (Mean = 22.13 years, SD = 2.95) completed study two. Both studies were temporally independent, with different participants in each study. Only one participant from study one also completed study two. Participant overlap was not a concern, as we aimed to temporally validate the ML models. The sample size was consistent with previous research (See^10^). All participants had normal or corrected-to-normal vision, and no neurological disorders, chronic pain disorders or acute pain at the time of participation. Participants were reimbursed £10 per hour for their time. Participants provided fully informed written consent at the beginning of both experiments. Both studies achieved ethical approval from the University of Liverpool Health and Life Sciences Research Ethics Committee. All methods in both studies were conducted in compliance with the Declaration of Helsinki.

### Pneumatic pressure stimulator

For both studies, tonic pain stimulation was delivered to the finger-nail bed of the left-hand index finger using a custom-built pneumatic pressure stimulator (Dancer Design, St. Helens, UK), as utilised in previous pain research^51^. The pneumatic stimulator consisted of a pneumatic force controller, which directed compressed air from an 11.1-L aluminium cylinder into the stimulator, which lowered a 1 cm^2^ probe to deliver the desired stimulation force. The stimulator was controlled using a LabJack U3 printed circuit board for interface. The pressure was limited to a maximum of 3.5 bar (9 kg/cm^2^) to prevent injury.

### Experimental procedure

#### Study one

Following the EEG cap fitting, participants were seated 1-m from a 19-inch LCD monitor inside a Faraday cage. Participants placed their left-hand index finger into an individualised mould that correctly positioned the finger underneath the stimulator probe. A thresholding procedure was employed to identify participants’ pain threshold and high pain intensity stimulus. Participants were verbally instructed to rate the pain intensity of each stimulus on an 11-point visual analogue scale (0–10) by using the mouse in their right hand to click the desired rating. On the rating scale, 0 reflected no sensation, 3 represented pain threshold and 10 reflected extreme pain. Participants were informed that any rating below 3 represented non-painful sensations. Following the instructions, a staircase thresholding procedure was implemented. The stimulus intensity was initialised at 0.5 bar pressure and incremented in steps of 0.2 bar (0.1 if preferred at higher levels) up to a maximum of 3.5 bar. The intensity that elicited repeated responses of 6 (± 1) on the 11-point scale on three successive trials was used as the high pain intensity stimulus. Moreover, the stimuli intensity that produced a repeated rating of 3 was determined as the pain threshold. Finally, an additional stimulus intensity was defined as two-thirds of the participant’s pain threshold stimulus intensity and reflected non-painful touch stimulation.

During the experiment, participants were requested to focus on a fixation cross, displayed on the monitor to minimise eye movements. Each trial consisted of the stimulus delivery and the post-stimulus rating. The stimuli delivery consisted of the rise time (time for the stimulation to increase from 0 bar to the desired intensity) followed by a 3-s hold time (duration the desired stimulus was delivered). For the rise time, the stimuli increased by 1/10th of the desired pressure every 0.1 s (to achieve the desired stimuli after 1-s). Subsequently, the stimulus intensity was maintained for 3-s before the probe was released, and a fixation cross was presented for a rest period of 5-s. Participants subsequently rated the pain intensity on a 101-point visual analogue scale, using the mouse in their right hand. The scale was anchored at 0, which reflected no sensation, and 100, which represented extreme pain. The rating phase continued until the participant successfully rated the stimuli. The rating phase was followed by a 2-s rest period and instructions for participants to place their finger back into the mould if they had removed it. Participants underwent a further 2-s rest period before progressing to the next trial.

The experiment contained three blocks, lasting approximately 15-min each, separated by intervals of 5–10 min. Forty trials with a minimum interstimulus interval (ISI) of 16-s were delivered per block, consisting of the three stimuli intensities. The stimuli were pseudo-randomised, such that no two consecutive trials consisted of the same intensity and that an equal number of stimuli were presented in each block. There were 13 trials of each of the two conditions and 14 trials of the remaining condition in each block, such that all stimuli conditions were delivered 40 times over the entire study. Consequently, a total of 120 stimuli were delivered in the experiment. Following the completion of all blocks, the EEG cap was removed, and participants were debriefed.

#### Study two

Study two used similar procedures to study one but consisted of different stimulation parameters. A 2 × 2 factorial design was employed with 4 conditions: low pain fast rise time, low pain slow rise time, high pain fast rise time, and high pain slow rise time. The low and high pain intensities were determined using the same thresholding procedure as study one. The high and low pain fast rise time conditions were identical to the stimulation in study one (1-s rise, 3-s hold). For the slow rise time conditions, the speed at which the probe lowered onto the left-hand index finger was reduced, increasing the rise time to three seconds. The stimuli increased from 0 bar to the desired intensity, in 1/30th increments of the desired stimuli every 0.1 s, until the desired intensity was reached and maintained for 3-s. After each stimulus, participants rated their pain on the same 101-point rating scale as study one.

Study two was comprised of three experimental blocks, lasting approximately 20-min each. Blocks were separated by 5–10-min intervals. The experiment consisted of 144 trials, with 48 trials with a minimum ISI of 16 s in each block. Blocks consisted of 12 trials of the four conditions, which were pseudo-randomised using similar randomisation as study one. On completion of the experiment, the EEG cap was removed, and participants were debriefed. Both experiments were delivered using PsychoPy2^52^.

#### EEG acquisition

EEG recordings were continuously obtained using a 129-channel EGI System (Electrical Geodesics, Inc., Eugene, Oregon, USA) and a sponge-based Geodesic sensor net. Net positioning was aligned with respect to three anatomical landmarks: two pre-auricular points and the nasion. Electrode-to-skin impedances were maintained below 50 kΩ for all electrodes throughout the experiment. A recording bandpass filter was set at 0.001–200 Hz, with the sampling rate set at 1000 Hz. Electrode Cz was set as the reference electrode.

#### EEG pre-processing

EEG pre-processing was performed using BESA 6.1 (MEGIS GmbH, Germany). Firstly, low- and high-pass filters were applied at 70 Hz and 0.5 Hz, respectively. Secondly, a notch filter of 50 ± 2 Hz was implemented. Oculographic and electrocardiographic artefacts were removed using principal component analysis (PCA)^53^. Additionally, electrode channels containing large artefacts were interpolated to a maximum of 10% of channels. None of the data in either study surpassed this threshold. Finally, the data were resampled to 256 Hz. Consequently, according to Shannon Sampling Theory, the theoretical maximum frequency that could be assessed was 128 Hz in this study (sampling rate/2)^54^. Although, more conservative measures recommend a minimum sampling rate of 2.5 times the maximum frequency of interest; resulting in a maximum frequency of approximately 102 Hz^55^.

Spectral analyses were conducted using MATLAB 2020a (The MathWorks, Inc., Natick, Massachusetts, USA) and EEGLAB 2021.1^56^. Firstly, power spectra density (PSD) was estimated using Welch’s method. The power spectra computation spanned − 4 to 6 s relative to the trial onset, in 1-s segments, shifted in 0.05-s increments. The data were smoothed using multi-taper Slepian sequences. Estimates of the PSD were computed between 1 and 70 Hz, with a resolution of 1 Hz. The relative band power change was calculated across every time point and frequency, in the entire epoch using the event-related desynchronisation (ERD) method^57^ (See Eq. 2). The estimate of ERD at each datapoint (e.g., A in the equation) is calculated by subtracting the mean PSD of the baseline period (− 3.5 to − 0.5; R), followed by a numerical transform to give relative change in power as a percentage value.

2 $$ERD \left( \% \right) = \left( {\frac{A - R}{R}} \right)*100$$

Negative ERD values represent decreases of band power in the active, relative to the baseline period, indicating cortical activation, while positive values reflect band power increases, known as event-related synchronisation (ERS). For the ML analysis, ERD data were collapsed across established frequency bands theta (4–7 Hz), alpha (8–12 Hz), lower beta (16–24 Hz), upper beta (25–32 Hz) and gamma (33–70 Hz). Topographical maps, to illustrate power changes from baseline to both low and high pain stimulation conditions of study one are reported in the supplementary material for illustrative purposes. ERD visualisation was conducted and reported following recommendations from previous research^57,58^.

### Classification procedure

Firstly, we identified the trials relating to low and high pain conditions. In the current study, high and low pain samples were determined by the stimulation intensity rather than the subjective rating, as this may ultimately serve as a proxy measure for subjective reporting for populations who cannot accurately report their pain intensity. Secondly, touch intensity trials from study one were removed as study two did not contain touch trials. EEG data from two participants in study one was heavily contaminated with artefacts. Both participants’ data were consistently contaminated with severe artefacts (e.g., muscle movement), which could not be resolved without exclusion. No threshold was used to determine exclusions in this instance, as it was evident from visual inspection that the data was not useable. Therefore, both participants were excluded, resulting in a final population size of 23. One participant was removed from study two due to corrupted data, which affected approximately 1/3 of the data. As a result, the final population was 14 in study two. All 14 participants from study two contributed to both external validation one and two, as both datasets were collected during the same session.

Candidate features were created using the single-trial time–frequency transformed data from study one. We computed 15 candidate features for ERD outputs in each specified frequency band which were calculated over the entire trial window [− 4 to 6 s] for all 128 electrodes, resulting in 9600 candidate predictors. The features were primarily descriptive statistics of the relative band power changes in each frequency band including the mean, mode, median, minimum, maximum, standard deviation, root mean squared, variance, skewness, kurtosis, absolute mean, Shannon entropy, log energy entropy, range and squared mean values for the time window of each trial. Candidate features used in this study were selected based on previous pain research^59,60^, which were calculated using MATLAB built-in functions where possible. Moreover, the features used in this study have been extensively explored in other research domains^61–64^. We opted to include this selection of different candidate features as, due to the complexity of EEG and ML, it is challenging to predict the effectiveness of the features and algorithms prior to modelling.

Due to neural variability and volatility of single-trial EEG^65–67^, missing values and outliers (values beyond three median absolute deviations) were replaced using linear interpolation. Interpolated values were calculated from neighbouring non-outlier data per condition using the *filloutliers* MATLAB function. Outliers were interpolated as they do not follow patterns, which hinders ML performance^68^. Additionally, outlier management is essential for EEG, as artefacts include non-neural activity^69^. The data were interpolated to maximise the dataset size, as larger datasets are less susceptible to overfitting^45^. Overall, less than 10% (M = 9.84%, SD = 0.55%) of the data were interpolated.

The features were scaled between 0 and 1 and univariate feature selection was employed to rank feature importance. We opted for a data-driven approach, meaning that all candidate features (e.g., all electrode locations and frequency bands) were evaluated during feature selection. Following feature ranking, a form of sequential feature selection was implemented to identify the optimal number of features. Here, the models were trained and evaluated using cross-validation with only one feature initially. Features were added sequentially until performance stabilised. Through this process, the highest-ranking 50 features were selected as this combination achieved near-optimal cross-validation performance without significantly increasing model complexity. The variables identified by the feature selection algorithm were distributed across various electrode locations and included features from frontal, central, and parietal regions. The electrode locations for all frequency bands assessed are displayed in the supplementary material (Figure S1). Moreover, the number of trials after pre-processing for both studies are presented in Table 1.

**Table 1:** Number of events per condition for each validation procedure.

Condition	Training and cross validation sets	External validation one set (identical stimuli)	External validation two set (different stimuli)	Total
Low pain	919	503	504	1926
High pain	897	504	504	1905
Total	1816	1007	1008	3831

ML was conducted using Scikit-learn, an open-source ML library written in Python, which offers efficient implementations of many ML algorithms^70,71^. We implemented an adaptive boosting algorithm (AdaBoost), linear discriminant analysis (LDA), logistic regression (LR), gaussian naïve Bayes (NB), random forest (RF), support vector machine (SVM), and an extreme gradient boosting algorithm (XGBoost) (see^14,15,72^ for overviews). Additionally, hyperparameter optimisation was performed on the cross-validation dataset using grid search, a common technique that assesses a fixed set of potential values for each hyperparameter and evaluates all possible combinations to identify the optimal configuration^73^. Grid search has been shown to improve ML performance over unoptimised parameters^73^, and previous research has implemented grid search^40,74^. The optimal hyperparameters (except for the NB, which does not require optimisation) are presented in Table 3 (see Discrimination and Calibration Results).

### Model evaluation

**Figure 1:**
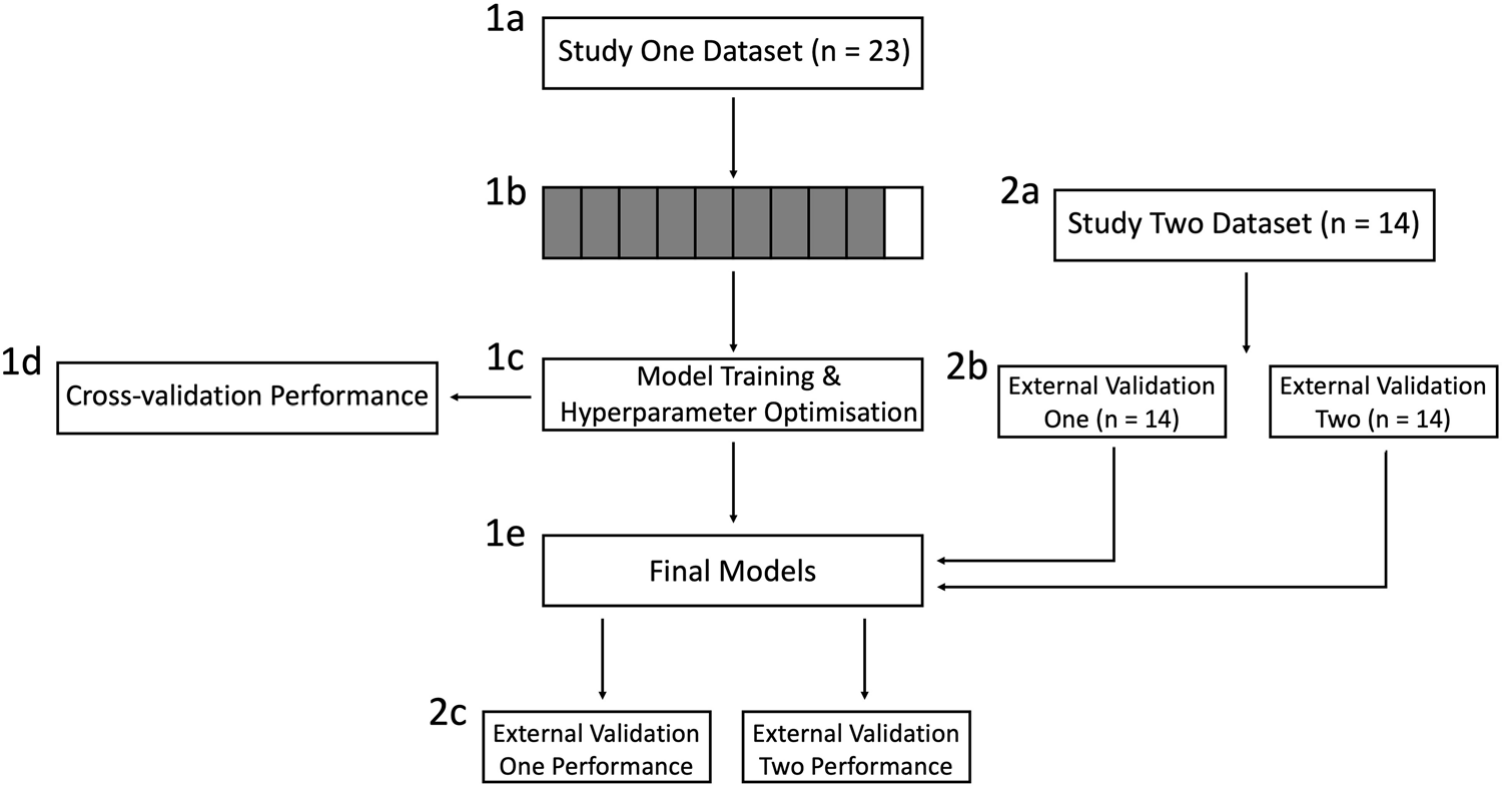
Flow chart of the classification pipeline. The final dataset from study one was cleaned, and features of interest were extracted (**1a**). The dataset, which was comprised of all 23 participants’ data, was split into 10 approximately equal folds (**1b**), with 9 folds used for training and 1 fold used for testing. Candidate models were then trained 10 times until all folds had been used for testing. During the training process, the hyperparameters of each model were optimised using grid search (**1c**). After training, the models’ cross-validation performance was examined (**1d**) and the final models and hyperparameters were selected based on the best cross-validation performance (**1e**). The dataset for study two was prepared using a similar pipeline (i.e., data cleaning) to study one, but was managed independently to prevent data leakage (**2a**). The dataset for study two was then split into external validation one and two, based on the trial types of the study (fast and slow rise) (**2b**). All 14 participants in study two contributed to both external validation datasets. Finally, the final models were tested separately on external validation one and two datasets, and model performance (discrimination and calibration) was assessed.

Cross-validation was performed using stratified k-fold validation, whereby the dataset is divided into *k* partitions, with one partition used for validation and the remaining for training. Each model is trained *k* times, with a different validation set at each iteration, meaning all data is used for validation^43,50,75^. Model performance is then averaged over all iterations. Stratified k-fold is advantageous over traditional k-fold as class distributions are preserved in each partition, rather than being random^50,75^. We set the value of* k* = 10^43^. The models were also assessed using a two-stage external validation procedure. For each validation, we computed accuracy, precision, recall, F1, AUC and brier scores to assess performance^76–79^ (See Supplementary Material for overviews). A flow chart of the classification procedure is presented in Fig. 1.

## Calibration assessment

We also assessed model calibration. Calibration assessment evaluates the agreement between the model’s prediction and the observed or reference value^50,79,80^. If a model predicts a 30% risk of an outcome being present, then the observed outcome frequency should be approximately 30 of 100 events^50,80,81^. For example, in a diagnostical context, in individuals with a predicted risk of *x%* for having a medical condition, *x* out of 100 individuals should have the condition^82^. Calibration is important for model evaluation but is rarely evaluated^10,83^. We assess calibration using calibration curves, whereby the predicted probability is plotted on the x-axis, and the true probability is plotted on the y-axis. Perfect calibration occurs when the predicted probabilities perfectly match the observed probabilities, which is represented by a 45° line in calibration curves. Comprehensive overviews of prediction model calibration assessment have been reported elsewhere^80,84^.

## Statistical analysis

Statistical analyses were conducted to investigate self-reported pain ratings for both studies. Firstly, a paired sample t-test assessed whether pain ratings differed between the low and high pain stimuli in study one. For study two, we assessed whether pain ratings differed between low and high stimuli and the fast and slow rise time conditions, using a 2 × 2 repeated measures ANOVA with the levels being stimuli intensity (low, high) and rise time (fast, slow). Statistical analysis was completed using IBM SPSS 27 (IBM Corp., Armonk, New York, USA).

## Results

### Behavioural pain ratings

Descriptive statistics for the behavioural pain ratings for both studies are presented in Table 2. A paired samples t-test demonstrated that subjective pain ratings in the high pain condition were significantly greater than those in the low pain condition in study one (*t* (22) = 12.71, *p* < 0.001, *d* = 2.65).

**Table 2:** Descriptive statistics (Mean ± standard deviation) for pain ratings across condition and study paradigm.

Condition	Low pain	High pain
**Study one**		
Cross-validation dataset (fast rise)	36.87 ± 13.44	62.65 ± 15.28
**Study two**		
External validation one dataset (fast rise)	50.51 ± 12.96	73.53 ± 10.61
External validation two dataset (slow rise)	47.22 ± 12.55	68.77 ± 9.83

Regarding study two, a 2 × 2 repeated measures ANOVA demonstrated a significant main effect of stimuli intensity on subjective pain ratings (*F *(1,13) = 53.91,* p* < 0.001, η_p_^2^ = 0.81), with pain ratings being significantly higher in the high pain conditions compared to the low pain conditions. Additionally, the analysis demonstrated a significant main effect of rise type on subjective pain ratings (*F *(1,13) = 14.94,* p* = 0.002, η_p_^2^ = 0.53), with subjective pain intensity being higher in the fast rise time conditions compared to the slow rise time conditions. Finally, the ANOVA demonstrated that there was no significant interaction between stimuli intensity and rise type on subjective pain intensity (*F *(1,13) = 1.25,* p* = 0.284, η_p_^2^ = 0.09).

### Discrimination and calibration results

**Figure 2:**
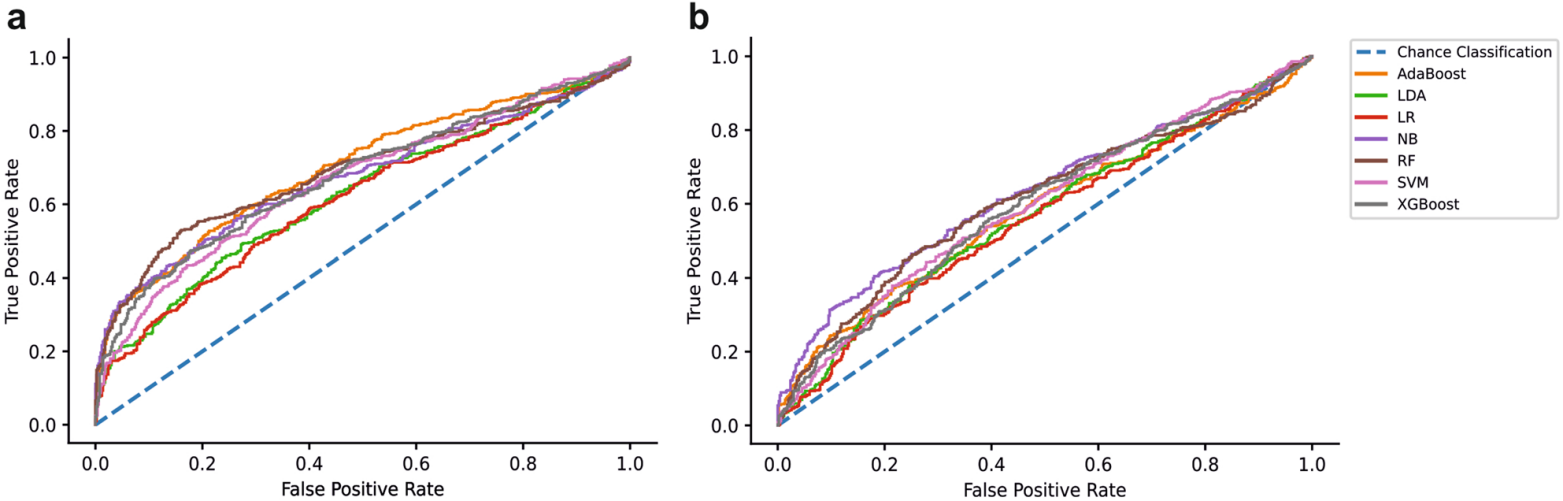
Discrimination results for both external validation stages. (**a**) ROC curve for all models assessed on the first external validation dataset. (**b**) ROC curve assessment on the second external validation dataset. The dotted blue line represents chance classification (a classifier with no skill) as a reference.

The classification performance metrics and optimal hyperparameters are reported in Table 2. The ROC curves for both external validation stages are presented in Fig. 2. In addition, the confusion matrices are reported in the supplementary material, allowing for the calculation of additional metrics, which may be of interest to readers and to those conducting meta-analyses.

The results can be segmented based on the type of validation performed. Regarding cross-validation discrimination, the results demonstrate that all the models perform better than chance on all metrics. The models achieved accuracies between 67.73 and 77.32% and AUCs between 0.7676 and 0.8644. Out of the seven models tested, four achieved accuracies greater than 70%. Moreover, the AdaBoost model achieved the best performance overall, recording the highest accuracy (77.32%) and AUC (0.8644) during cross-validation.

Regarding external validation one, the results demonstrate that the models performed better than chance on most of the performance metrics. The accuracy of the models ranged from 58.99 to 68.32%, whilst the AUC ranged from 0.6170 to 0.6995. Here, six out of the seven models achieved accuracies greater than 60%. Moreover, the RF model achieved the highest accuracy (68.32%), whilst the AdaBoost model recorded the best AUC (0.6995) on the first external validation dataset. However, it must be noted that the AdaBoost model only marginally exceeded the RF at this validation stage, with the RF achieving an AUC of 0.6910.

Lastly, for the discrimination results, the models achieved accuracies between 54.76 and 60.42% and AUCs ranging from 0.5615 to 0.6288 on external validation two. Two models (RF and NB) achieved accuracies greater than 60%. In line with the first external validation, the RF achieved the best accuracy (60.42%) on the second validation dataset, whilst the NB algorithm achieved the greatest AUC (0.6288).

**Figure 3:**
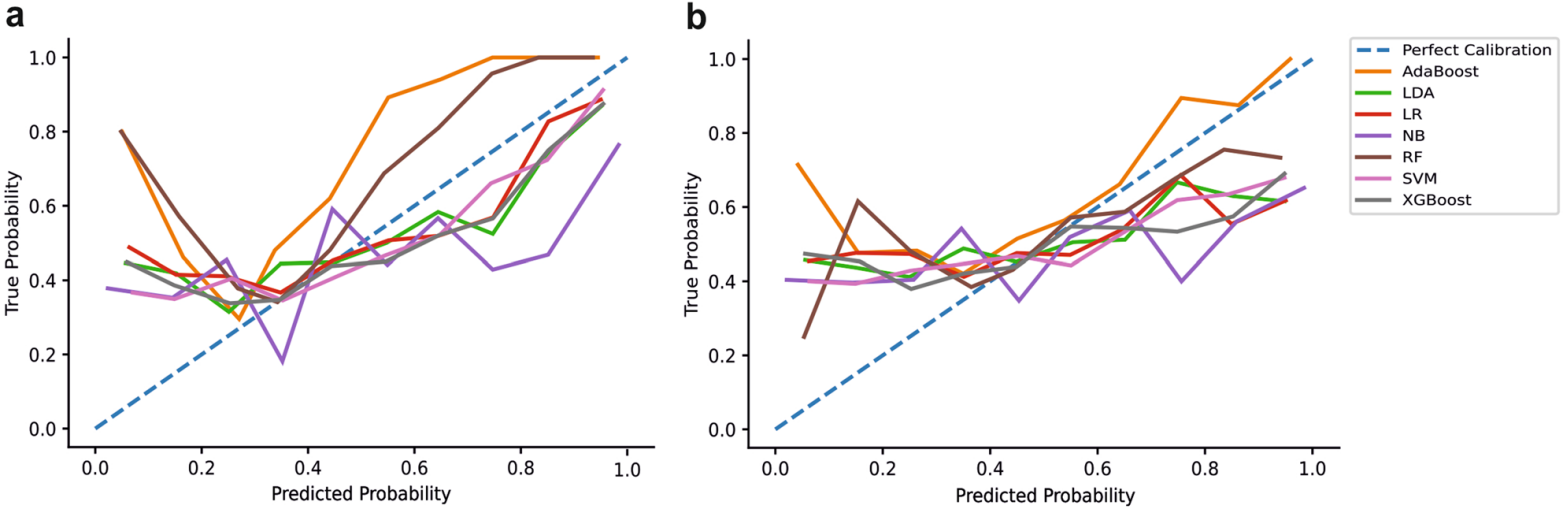
Calibration results for both external validation stages. (**a**) Calibration curve for all models assessed on the first external validation dataset. (**b**) Calibration curve for the second external validation dataset. The blue dotted line (45°) represents perfect calibration (complete agreement between predicted and observed probabilities). When the colour line is above the reference, the model underestimates the true probability, whilst the model overestimates probabilities when the line is below the reference line.

Finally, we also assessed the calibration of the models. The calibration plots for all models across both external validation stages are presented in Fig. 3. Regarding the interpretation of the calibration curves, if the model line is above the reference line, it suggests that the model is underestimating the probability of the incidence, whilst the inverse insinuates that the model is overestimating the incidence prevalence. Finally, the Brier score provides a metric of the disparity between predicted and true outcome probabilities is reported in Table 3.

**Table 3:** Classification performance metrics for cross validation and both external validation procedures.

Model	Optimal parameters	Cross validation (Mean ± SD)		External validation one		External validation two	
AdaBoost	Learning rate = 0.1, Number of estimators = 2500	Accuracy	0.7732 ± 0.0374	Accuracy	0.6385	Accuracy	0.5595
		AUC	0.8644 ± 0.0199	AUC	0.6995	AUC	0.5823
		Brier	0.2450 ± 0.0011	Brier	0.2473	Brier	0.2488
		F1	0.7596 ± 0.0469	F1	0.6459	F1	0.5681
		Precision	0.7983 ± 0.0538	Precision	0.6336	Precision	0.5573
		Recall	0.7302 ± 0.0717	Recall	0.6587	Recall	0.5794
Linear discriminant analysis	Shrinkage = 0.4, Solver = Least squares	Accuracy	0.6965 ± 0.0249	Accuracy	0.6008	Accuracy	0.5625
		AUC	0.7707 ± 0.0307	AUC	0.6248	AUC	0.5724
		Brier	0.2007 ± 0.0135	Brier	0.2609	Brier	0.2888
		F1	0.6809 ± 0.0450	F1	0.5630	F1	0.5127
		Precision	0.7114 ± 0.0473	Precision	0.6226	Precision	0.5786
		Recall	0.6665 ± 0.1042	Recall	0.5139	Recall	0.4603
Logistic regression	C = 1.0, Penalty = Lasso (L1), Solver = LibLinear	Accuracy	0.6910 ± 0.0301	Accuracy	0.5899	Accuracy	0.5476
		AUC	0.7676 ± 0.0283	AUC	0.6170	AUC	0.5615
		Brier	0.1990 ± 0.0108	Brier	0.2544	Brier	0.2793
		F1	0.6793 ± 0.0391	F1	0.5663	F1	0.5043
		Precision	0.7024 ± 0.0548	Precision	0.6013	Precision	0.5577
		Recall	0.6687 ± 0.0856	Recall	0.5357	Recall	0.4603
Naïve bayes	-	Accuracy	0.7137 ± 0.0432	Accuracy	0.6395	Accuracy	0.6012
		AUC	0.8011 ± 0.0362	AUC	0.6746	AUC	0.6288
		Brier	0.2382 ± 0.0378	Brier	0.2978	Brier	0.3437
		F1	0.6806 ± 0.0807	F1	0.6142	F1	0.5830
		Precision	0.7532 ± 0.0513	Precision	0.6613	Precision	0.6109
		Recall	0.6377 ± 0.1339	Recall	0.5734	Recall	0.5575
Random forest	﻿Criterion = Entropy, Maximum depth = 10, Maximum features = Log_2_, Number of estimators = 350	Accuracy	0.7318 ± 0.0556	Accuracy	0.6832	Accuracy	0.6042
		AUC	0.8129 ± 0.0392	AUC	0.6910	AUC	0.6088
		Brier	0.2008 ± 0.0100	Brier	0.2217	Brier	0.2409
		F1	0.6748 ± 0.0961	F1	0.6216	F1	0.5481
		Precision	0.8315 ± 0.0757	Precision	0.7729	Precision	0.6385
		Recall	0.5830 ± 0.1253	Recall	0.5198	Recall	0.4802
Support vector machine	C = 1.0, Gamma = 0.1, Kernel = RBF	Accuracy	0.6773 ± 0.0189	Accuracy	0.6187	Accuracy	0.5645
		AUC	0.7844 ± 0.0226	AUC	0.6647	AUC	0.5956
		Brier	0.1927 ± 0.0084	Brier	0.2369	Brier	0.2653
		F1	0.6669 ± 0.0454	F1	0.6265	F1	0.5675
		Precision	0.7279 ± 0.0515	Precision	0.6145	Precision	0.5636
		Recall	0.6298 ± 0.1013	Recall	0.6389	Recall	0.5714
XGBoost	Column sample by tree = 1.0, Gamma = 1.5, Maximum depth = 2, Minimum child weight = 1, Subsample = 1.0	Accuracy	0.7527 ± 0.0337	Accuracy	0.6246	Accuracy	0.5754
		AUC	0.8362 ± 0.0270	AUC	0.6770	AUC	0.5931
		Brier	0.1657 ± 0.0134	Brier	0.2336	Brier	0.2756
		F1	0.7282 ± 0.0591	F1	0.6205	F1	0.5737
		Precision	0.7922 ± 0.0405	Precision	0.6280	Precision	0.5760
		Recall	0.6845 ± 0.1019	Recall	0.6131	Recall	0.5714

## Discussion

This study represents the first successful attempt to externally validate ML to discriminate between high and low pain intensity using EEG. We hypothesised that all ML algorithms would achieve greater than chance performance (≈50%) on (1) cross-validation, (2a) external validation one (same stimulation parameters as training data), and (2b) external validation two (different stimulation parameters to training data). Our results demonstrated that all models surpassed chance performance, achieving accuracies of up to 78%, 69% and 61% on cross-validation and external validation one and two, respectively. The RF model demonstrated the highest accuracy on both external validation stages. Overall, the findings support our hypothesis. This study is the first to demonstrate that ML and EEG can be effectively combined for binary classification of pain intensity with accuracies approaching 70% using external validation. Moreover, the second external validation confirms the robustness of the results, demonstrating that ML can accurately classify experimentally induced pain intensity using different stimulation parameters, which is imperative for translation when minor variations in the nature of pain should not invalidate the algorithm. Therefore, this study advances the field, correcting widespread limitations and providing the first rigorous and generalisable estimates of the effectiveness of ML and EEG for pain intensity classification.

Our findings support previous literature demonstrating that subjective pain intensity can be accurately classified using EEG and ML^9,10^. The cross-validation performance in this study is comparable to previous research^10^. Previous attempts to classify low and high pain intensity from EEG have produced comparable results, with accuracies ranging between 62 and 89.58%^35–40^. Similar research successfully classified 10-classes of pain intensity using a RF model and multichannel EEG^41^. Our findings support the existing literature, as both studies demonstrate the importance of using a diverse array of frequency bands to achieve optimal classification performance. In addition, Huang and colleagues^39^ developed models using single-trial laser-evoked potentials, capable of accurately classifying low and high pain for both within-subject and cross-subject predictions. Alternative neuroimaging (e.g., fMRI) approaches also demonstrate promise for pain outcome prediction^9^. For example, the neurologic signature of pain demonstrated 93% sensitivity and specificity in discriminating between no pain and pain conditions in a novel sample^17^. Overall, the previous research demonstrates the potential of neuroimaging and ML for pain intensity classification. However, EEG may prove to be the optimal method after further validation, due to its accessibility, ease of use, and low cost^85,86^, which offers potential for the method to be used in a more diverse array of use cases.

Whilst our results are comparable to the best-performing models of the existing literature (e.g., classifying better than chance), it must be noted that several models reported across all studies had reduced performance, demonstrating the importance of careful evaluation. Moreover, the literature is comprised of positive results, which may be a result of publication bias and therefore should be carefully interpreted. In addition, previous research assessed model performance using only internal validation methods (e.g., cross-validation), meaning that overfitting and generalisability had not been sufficiently evaluated^10^. Therefore, the novelty and impact of the present research stem from the extensive external validation. Presently, the clinical potential of ML and EEG for pain prediction has likely been overestimated^45,48,49^ and significant developments are required before the clinical potential can be accurately assessed. However, although our results are modest, the current study extends upon previous research, demonstrating that ML and EEG can accurately classify novel samples which provides more robust evidence for the clinical utility of ML.

Beyond EEG, alternative proxy pain measures have been proposed (e.g., behavioural assessments). Many behavioural approaches rely on facial expressions (e.g., PACSLAC^87^ or ML techniques^88^), which is time-consuming^88^ and can be erroneous in individuals with dementia (e.g., Lewy Body)^89^, Parkinson’s disease^90^, or facial paralysis (e.g., locked-in syndrome)^91^, as well as children who can suppress pain expressions^92^. EEG and ML may provide effective pain assessment in these challenging conditions. Pain-related neural activity is observable across populations (e.g., infants)^93^ and should not be affected by intentional suppression. Therefore, EEG-ML methods could become useful adjunctive pain assessment tools, specifically in situations that have previously proved challenging.

EEG-ML approaches may also prove advantageous over other pain biomarker techniques. Physiological measurements including heart rate variability (HRV), electrodermal activity (EDA), and pupillometry demonstrate potential^94^. However, such approaches also exhibit significant limitations, which often result in reduced effectiveness in certain populations (e.g., paediatric postoperative patients^95^). Moreover, alternative neuroimaging techniques remain promising (e.g., fMRI)^9,17^. However, many neuroimaging techniques are impractical for widespread clinical implementation, due to financial and infrastructure restrictions^96^. EEG is inexpensive compared to fMRI and can be easily implemented in a multitude of settings (e.g., doctor’s office) using dry or mobile EEG^85,86,97,98^. Furthermore, EEG can be used during surgery^99^ and can also be further simplified using a single electrode^100^. Taken together, EEG may be advantageous over other methods, demonstrating diverse utility in clinical settings.

The findings from this study also highlight the importance of external validation, as cross-validation metrics did not consistently reflect external validation metrics, which challenges previous EEG and ML research. It is established that ML performs better on data from the same cohort (internal validation) when compared to novel samples (external validation)^46,47^. Consequently, cross-validated metrics are potentially biased and not representative of prediction errors^44,45,47^. In this study, the AdaBoost model achieved the best cross-validation metrics but performed worse than the RF on both external validations. As the RF performance only reduced minimally during external validation, we have increased confidence that the model has learned pain-related information, rather than fitting random noise. Furthermore, small reductions in performance when progressing from cross-to-external validation procedures are common and should not invalidate the model’s clinical utility^46,47,101^. Given the subjective nature of pain^1,4^ and variability of neural activity (e.g., single-trial EEG)^65–67^, a reduction of only 5% demonstrates the RF’s robustness, providing evidence for the clinical potential of this approach. Overall, our research emphasises that failing to include external validation in experimental paradigms reduces clinical interpretation^48,49^ and should be avoided in future research. We also recommend caution when interpreting research that only reports cross-validation, to avoid presenting over-optimistic results, which could hinder future efforts towards clinical translation.

Models that are not sufficiently evaluated are potentially damaging to the clinical utility of ML and EEG. A biased algorithm risks that patients could receive sub-optimal care (e.g., under-treatment), which has significant dangers^48,102^. Indeed, ML models failing due to biases are common and may be overlooked without sufficient validation (e.g., skin markings in dermoscopic images inflating the probability of an input being classified as a melanoma using a convolutional neural network)^103^. Such biases may render the algorithm useless. Therefore, our research provides a foundational development toward clinical translation and paves the way for improved standards in ML-EEG studies for pain classification.

ML and artificial intelligence (AI) are rapidly advancing society (e.g., route planning and self-driving vehicles), but successful medical applications are rare^104,105^. Clinical translation requires significant developments spanning external validation to dissemination^96^. Whilst our best model is an important initial development, the performance is not currently clinically applicable. Further external validation is imperative, particularly through international multi-centre collaborations^9,10,96^ to demonstrate clinically relevant performance. This would evaluate algorithms using larger, more diverse samples, allowing for greater confidence that the algorithm is not biased by dataset idiosyncrasies, which are specific to a single lab’s apparatus or procedures^85^. Moreover, progression to research in clinical populations which attempts to classify clinical rather than experimental pain is critical to establish the clinical utility of the method. Subsequently, the clinical translation pipeline should be carefully navigated. Real-world and utility assessments (e.g., randomised controlled trials) should ensure the algorithm is useful to clinicians^96,105^. Moreover, feasibility, safety, ethical and acceptability considerations will be essential to establish appropriate deployment standards to limit risk before dissemination^85,96,105^. However, before attempting these stages significant further research is required. Establishing a substantial body of external validation research, including multi-centre collaborations must be the primary objective. The long-term future of clinical ML applications for pain is contingent on the collective research community successfully addressing the clinical translation stages.

The current study has several limitations. Firstly, the calibration assessment demonstrated that the predicted probabilities were not consistently representative of the true probabilities. Consequently, the clinical potential of the findings at this early stage should be interpreted with caution. Imperfect calibration is suggestive of potential overfitting, reducing validation performance due to the idiosyncrasies in the training data^80^. However, given the volatility of neural activity^65–67^, it is to be expected that the models capture some random noise. As calibration is rarely assessed^10,83^, future research should aim to assess and improve model calibration (e.g., Platt scaling)^84^. Moreover, whilst this study consists of two temporally independent datasets, our overall sample size is relatively small, which reduces the confidence in the results. For ML to exhibit clinical relevance, a larger, more diverse sample is required. Future research should increase sample sizes to provide more robust conclusions, which would offer substantial further evidence for clinical translation. In addition, there was some overlap between the samples, with one participant contributing to both the development and validation samples. Future research should avoid participant overlap, or specifically explore the differences between within—and cross-subject prediction. However, in the current study, both samples were temporally independent and consisted of different experimental paradigms. Therefore, participant overlap is unlikely to significantly affect the results. Moreover, although the sampling rate in this study was sufficient (sampling rate > 2.5 times the maximum frequency analysed) to retrieve gamma band frequencies and avoid aliasing issues^55^, future research should maximise the sampling rate to ensure that the highest frequencies are precisely sampled.

The current study predicted stimulation intensity rather than subjective intensity, as this may ultimately serve as a better proxy method for individuals who cannot self-report their pain. However, on a trial level, there were a few instances where a low-intensity stimulus produced a high subjective response and vice-versa. Consequently, such trials may have hindered the learning algorithms’ performance. Future research should investigate both subjective pain intensity and stimulus intensity. Additionally, it is possible that EEG signals used in the classification were not pain-specific, which should be explored in further research. Research has suggested that EEG responses to pain may be more directly related to stimulus saliency rather than pain perception^106^. Moreover, whilst classifying discrete pain classes has clinical potential, predicting parametric outcomes would improve the impact of the research. The ability to accurately predict subjective pain intensity to a finer resolution would increase clinical utility. Therefore, future research should externally validate regression models to demonstrate greater clinical relevance. Concurrent attempts to improve binary classification performance are also warranted before clinical translation. Finally, although the models in this study outperformed chance, we cannot definitively state that the models are exclusively reflective of neural processing. EEG signals can often contain non-brain responses e.g., muscle movements^107^, which could affect the results. Many of the features were from electrodes located over feasible brain regions and not exclusively from those electrodes most commonly impacted by movement artefacts such as peripheral sites^107^, which provides confidence in the results. Moreover, model performance generalised to two external validation datasets, which included different experimental pain stimulation. Therefore, we can reasonably suggest that pain-related brain information was the predominant contributor to accurate classification. However, despite thorough artefact correction, residual non-brain activity may be present in the EEG signal. Whilst our artefact correction procedure is extensively validated, it is possible residual non-brain activity may still contribute to the features and classification. For example, whilst similar research has used prefrontal theta as a feature for pain classification^40^, we cannot rule out the possibility that residual oculographic (e.g., saccades) or facial muscle movements may also contribute to the EEG data in the present study. Therefore, we propose that the importance of the frontal theta features should be interpreted with caution. Future research should aim to explore the role of non-brain responses on EEG pain classification using additional techniques such as the characterisation of electromyographic (EMG) signals or concurrent evaluation of facial expressions. In addition, future research should investigate the impact of different pre-processing procedures on pain classification performance, with the goal to develop standardised, reproducible pre-processing.

## Conclusion

This research study is the first to demonstrate that ML and EEG can be used in tandem to discriminate between low and high pain intensity using a comprehensive two-stage external validation paradigm. Our best-performing model (RF) classified low and high pain with around 70% accuracy on external validation with matched stimulation and around 60% with different experimental pain stimuli. The results presented here are a significant development for the research field, as we begin to address limitations that have hindered clinical interpretation in the past. Consequently, this study provides the current best estimates of the effectiveness of ML and EEG for pain intensity classification. Future research should strive to build on the work presented here by consistently externally validating models, before progressing to multi-centre validation studies. Overall, the current study demonstrates the potential of ML and EEG for successful pain intensity prediction and provides the first robust estimates of ML generalisability which have eluded all previous research in this field.

## Supplementary Information

Supplementary Information.

## Data Availability

The datasets generated and/or analysed during the current study are available in the OSF repository, https://osf.io/uqt9z/.
