# Supplementary material for: External validation of binary machine learning models for pain intensity perception classification from EEG in healthy individuals
Source: Sci Rep. 2023 Jan 5;13:242. doi: 10.1038/s41598-022-27298-1 (PMC9816165; doi:10.1038/s41598-022-27298-1)
Supplement: Supplementary file 1 — Supplementary Information. [file 41598_2022_27298_MOESM1_ESM.pdf]

External validation of binary machine learning models for pain intensity perception classification from EEG in healthy individuals

Supplementary Material

Tyler Mari[1*], Oda Asgard[1], Jessica Henderson[1], Danielle Hewitt[1], Christopher Brown[1], Andrej Stancak[1], Nicholas Fallon[1]

[1]Department of Psychology, Institute of Population Health, University of Liverpool, Liverpool, UK

Tyler Mari, BSc, MSc, Department of Psychology, University of Liverpool, 2.21 Eleanor Rathbone Building, Bedford Street South, Liverpool L69 7ZA, UK. E-mail: Tyler.Mari@liverpool.ac.uk. ORCID: 0000-0001-9062-5175

Methods

Model Evaluation

The primary measures of discrimination in the current study were the AUC and accuracy. The AUC measures the model's overall performance, which is the ability of the algorithm to correctly discriminate between low and high pain across different classification thresholds. An AUC of 0.5 represents chance discrimination, whilst an AUC of 1 represents perfect discrimination. Moreover, accuracy assesses the overall effectiveness of the algorithm and represents the number of correctly classified events over the total number of events. Precision measures the ratio of correctly labelled positive events across all positive predictions. In contrast, recall assesses the ratio of true positive cases correctly identified. F1 represents the harmonic mean of recall and precision. For accuracy, precision, recall and F1, outputs of 1 demonstrate perfect predictions, whilst 0.5 represents chance performance for binary classification (note, classification metrics, excluding AUC, are reported as a percentage in-text for improved readability). Finally, the Brier score measures the mean squared error of the probability prediction. Here, 0 represents perfect performance and 1 reflects the worst theoretical performance. The Brier score was assessed as it is affected by discrimination and calibration, which is advantageous over other metrics. Equations (1) to (5) provide mathematical descriptions of the metrics.

$$Accuracy = \frac{tp + tn}{tp + fp + tn + fn} \tag{1}$$

$$Precision = \frac{tp}{tp + fp} \tag{2}$$

$$Recall = \frac{tp}{tp + fn} \tag{3}$$

$$F1 = \frac{2tp}{2tp + fp + fn} \tag{4}$$

Where tp, tn, fp, fn represent the number of true positives, true negatives, false positives, and false negatives, respectively.

$$Brier\ Score = \frac{1}{n}\sum_{i=1}^{n}(p_i - o_i)^2 \tag{5}$$

Where n is the number of samples, p_i *is the probability prediction and* o_i *is the outcome event.*

Results

ERD/S

To provide an overview of the neural characteristics of pain, we provide topographical maps demonstrating the difference between high and low pain conditions. It is important to note that the ML classification process is separate from the visualisation, which is explained hereafter. Figure S1 shows the time-frequency changes during rest (-3.5 – -2.5 s relative to the onset of stimulation) and the active period (1 – 2 s relative to the onset of stimulation; representing a period of maximum pressure level following the completion of stimulation rise time) for study one. Topographic plots demonstrating relative band power changes in frequency bands Theta (4 – 7Hz), Alpha (8 – 12Hz), Lower Beta (16 – 24Hz), Upper Beta (25 – 32Hz), and Gamma (33 – 70Hz) are shown. The left pair of columns represent rest and active periods for the low pain condition, whilst the right pair of columns represent the high pain condition. During both low and high pain stimulation conditions, theta-band ERS was evident over anterior frontal regions. In the high pain condition, lateralised ERS over right and left temporal electrodes was also observed in theta frequency range (Figure S1 **A**). Importantly, strong bilateral ERD in the alpha band was observed over sensorimotor regions in both low and high pain conditions (Figure S1 **B**) with visibly stronger alpha ERD present in high-pain condition. Bilateral ERD was also evident in both lower and upper beta bands over sensorimotor regions for both pain intensity conditions. ERD is comparatively weaker for upper beta compared to lower beta (Figure S1 **C/D**). The bilateral ERD observed with painful stimulation in alpha and beta bands is consistent with previous research [1]. Finally, for Gamma band changes, we identified bilateral ERD across temporal-parietal regions and ERS over anterior frontal electrodes for both low and high pain conditions (Figure S1 **E**).

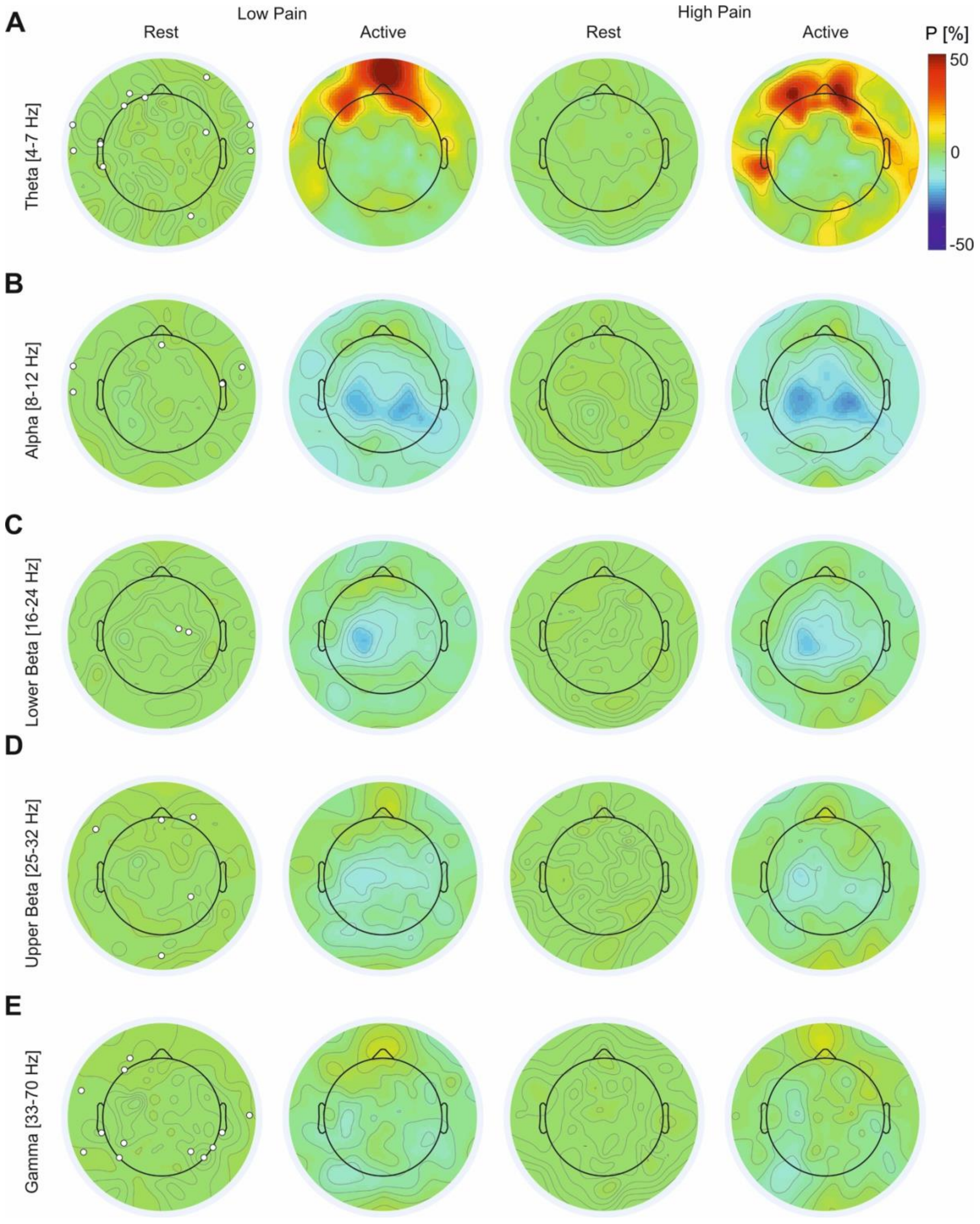

Figure S1. Grand average band power changes during rest (-3.5 s – -2.5 s) and during active pressure stimulation (1 s – 2 s) from study one. The trial period spanned from -4 s to 6 s relative to trial onset, with a baseline from -3.5 s to -0.5 s. The active period for visualisation was selected in line with previous recommendations [2,3] and reflected 1 s of continued pressure after the stimulator reached the desired intensity level. Topographic maps show the band power changes in low and high pain intensity conditions and from rest to active

periods in Theta (**A**), Alpha (**B**), Lower Beta (**C**), Upper Beta (**D**), and Gamma (**E**) for study one. P = percentage power change from baseline. The white circles on the low pain rest plots represent the electrode locations of the features used in the ML models.

Confusion Matrices

The confusion matrices for all models and external validation assessments are presented in Table S1.

Table S1. Confusion matrices for all models for both external validations.

Model	**External Validation One**		**External Validation Two**	
	Predicted Low Pain	Predicted High Pain	Predicted Low Pain	Predicted High Pain
AdaBoost				
Low Pain	311	192	272	232
High Pain	172	332	212	292
LDA				
Low Pain	346	157	335	169
High Pain	245	259	272	232
LR				
Low Pain	324	179	320	184
High Pain	234	270	272	232
NB				
Low Pain	355	148	325	179
High Pain	215	289	223	281
RF				
Low Pain	426	77	367	137
High Pain	242	262	262	242
SVM				
Low Pain	301	202	281	223
High Pain	182	322	216	288
XGBoost				
Low Pain	320	183	292	212
High Pain	195	309	216	288

Supplementary References

1. Ploner M, Gross J, Timmermann L, Pollok B, Schnitzler A. Pain Suppresses Spontaneous Brain Rhythms. *Cereb Cortex*. 2006;16(4):537-540. doi:10.1093/cercor/bhj001
2. Pfurtscheller G, Aranibar A. Event-related cortical desynchronization detected by power measurements of scalp EEG. *Electroencephalogr Clin Neurophysiol*. 1977;42(6):817-826. doi:10.1016/0013-4694(77)90235-8
3. Pfurtscheller G, Aranibar A. Evaluation of event-related desynchronization (ERD) preceding and following voluntary self-paced movement. *Electroencephalogr Clin Neurophysiol*. 1979;46(2):138-146. doi:10.1016/0013-4694(79)90063-4
